# Clinical Midwifery; with the Histories of Four Hundred Cases of Difficult Labour

**Published:** 1843-01

**Authors:** 


ART. III.—*Clinical Midwifery; with the Histories of Four Hundred Cases of Difficult Labour.* By ROBERT LEE, M.D. F.R.S.—*London,* 1842. 12mo, pp. 224.

THIS little work consists mainly of a series of reports published some time ago in the Medical Gazette, a fact which should have been noticed. These reports, we are told by the author, "comprise the most important practical details of all the cases of difficult parturition which have come under his observation during the last fifteen years; and they are now published in the hope that they may be found to illustrate, confirm, or correct the rules laid down by systematic writers for the treatment of difficult labours." Works of this sort, from men of ability and learning, are always valuable and useful; and that Dr. Lee possesses both these requisites is well known to the profession at large. We think that if the author had given, in a condensed form, the practical conclusions deducible from these cases, in a continuous series of aphorisms, the small volume would have been found of more use to the practitioner. As it is, it deserves the attention of all engaged in the line of practice to which it relates.

